# Sparse modeling of chemical bonding in binary compounds

**DOI:** 10.1080/14686996.2019.1697858

**Published:** 2019-11-27

**Authors:** Yosuke Kanda, Hitoshi Fujii, Tamio Oguchi

**Affiliations:** a Institute of Scientific and Industrial Research, Osaka University, Osaka, Japan; b MaDIS-CMI2, National Institute for Materials Science, Tsukuba, Japan

**Keywords:** Sparse modeling, machine learning, chemical bonding, binary compounds, 404 Materials informatics / Genomics

## Abstract

A sparse model for quantifying energy difference between zinc-blende and rock-salt crystal structures in octet elemental and binary materials is constructed by using the linearly independent descriptor-generation method and exhaustive search, following the previous work by Ghiringhelli et al. [Phys Rev Lett. 2015;114:105503]. The obtained simplest model includes only atomic radius information of constituent atoms and its physical meaning is interpreted in relation to van Arkel-Ketelaar’s triangle for classifying chemical bonding in binary compounds.

## Graphical Abstract

**Figure:**
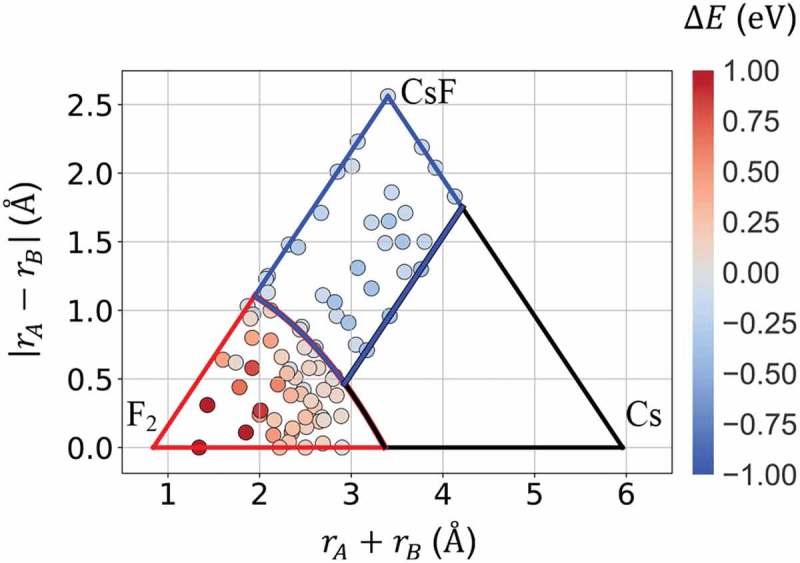

## 1. Introduction

Recently, data-intensive scientific discovery and design have been the focus of great attention for the acceleration of research and development in materials science, being widely called materials informatics (MI). The major aims of MI are the exploration of new materials with desired properties, the optimization of existing materials for particular performances, and the understanding of underlying physical mechanisms for further development. Generally, if one demands high predictability for a model constructed by data-science machine-learning techniques, complicated methods using non-linear models such as kernel ridge regression [1], neural network [2], and random forest [3] are appropriate, though their interpretation becomes troublesome because of the non-linearity involved in the modeling. On the other hand, simple modeling such as linear regression with interpretable descriptors is suitable for extracting intuitive understanding from materials data at the sacrifice of its predictability to a certain degree. Sparse modeling [4] is the statistical learning technique to realize such a simple model by the selection and reduction of the descriptors assumed.

A pioneering work with the use of the sparse modeling for materials properties was reported by Ghiringhelli et al. [5]. Total energy differences between zinc-blende and rock-salt crystal structures obtained by density-functional-theory (DFT) calculations for 82 elementary and binary semiconductors AB are modeled with the least absolute shrinkage and selection operation (LASSO) [6] and exhaustive search techniques within the linear regression modeling. They have succeeded to construct simple models with a small number of descriptors at relatively high predictability. The key to success can be found in the construction of the descriptors. They first assumed several basic descriptors such as ionization potential, electron affinity, and some DFT atomic data for constituent atoms and then operated them to get higher-order descriptors with multiplication, division, and functionalization up to the order of thousands. The LASSO technique is utilized to reduce the number of descriptors to tens by statistical procedures and error evaluations. Finally, an exhaustive search is used to extract the most important descriptors for a given number of descriptors among them. Nevertheless, the obtained model is still far understandable with physical intuitiveness because of complicated functions of several basic descriptors.

In this study, we aim to construct a simpler and interpretable model for the same problem as that Ghiringhelli and coworkers attacked. Our idea is two folds: one is the symmetrization of basic descriptors for the permutation of constituent elements A↔B and the second is the high-order operation of basic descriptors without using complicated functions like exponential. Also, regression trials with a single basic descriptor will be carried out. During the high-order descriptor operations, collinearity problems (including multicollinearity and near multicollinearity) often take place because of strong dependency between the generated higher-order descriptors by products of descriptors. The linear independent descriptor generation (LIDG) method recently proposed by us [7] is adopted to remove those collinearity problems if they happen. Our models are found to be as simple as the previous models, without utilizing complicated descriptors and able to quantitatively classify the chemical bonding in binary compound systems.

## 2. Methods

### 2.1. Target variables

The target variables prepared by Ghiringhelli et al. [5] are used for the construction of modeling in this study. Namely, total energy differences between zinc-blende (ZB) and rock-salt (RS) type structures calculated for 82 octet elementary and binary compounds AB with main-group elements as

(1) ΔE=E(RS)−E(ZB).

The data used for the present regression are listed in Appendix A. To confirm the precision of the target data, total energies of the 82 systems with ZB and RS structures are recalculated by using the all-electron full-potential linearized augmentation plane-wave method implemented in our HiLAPW code [8] and the root-mean-square errors are 7meV/atom in the total-energy difference and 0.009Å in the equilibrium lattice constant.

### 2.2. Descriptors

Ghiringhelli et al. [5] distinguished the constituent elements A and B according to the size of electronegativity. However, permutation of A and B leads to no physical change in the system at the equiatomic condition and models constructed should be symmetric by the permutation. In the present study, we generate descriptors as follows:

1. Prepare basic descriptors $x_{A}$ and $x_{B}$ for constituent atoms A and B on the basis of our intuition.
2. Symmetrize them by permutation A↔B and add their inversion, being called first-order descriptors.
3. Generate high-order descriptors by multiplication of the first-order descriptors.
4. Remove multicollinearity and near multicollinearity by erasing the irrelevant descriptors
5. Iterate to generate the high-order descriptor generation and to reduce collinearity problems, if needed.

Concerning the basic descriptors, easily obtainable physical quantities could appeal our intuition to construct interpretable models. Atomic radius, ionization potential, electron affinity, and electronegativity are adopted in this study and tabulated in Appendix A. As for the symmetrization and inversion, we consider the following operations as

(2) $$x_{A}+x_{B},|x_{A}-x_{B}|,|x_{A}+x_{B}|,\frac{1}{x_{A}+x_{B}},\frac{1}{|x_{A}+x_{B}|}.$$

The high-order descriptors are generated by multiplication of the first-order descriptors. From m first-order descriptors, ${\;}_{m}H_{n}{=}_{m+n-1}C_{n}$ n th-order descriptors can be constructed. As mentioned above, every time high-order descriptors are generated, multicollinearity and near multicollinearity are removed by the LIDG method [7]. Here, multicollinearity is a linear dependency between descriptor vectors. Such a linear dependency often occurs when higher-order descriptor generations are performed. The existence of the linear dependency means that Xc=0 has non-trivial solutions c, where $X=[x_{1},x_{2},...,x_{p}]$ is a descriptor matrix (design matrix) with descriptor vector $x_{i}$ as the columns. p is the number of descriptors. Thus, to find multicollinearity, all non-trivial solutions of Xc=0 should be found. Fortunately, the non-trivial solutions can be easily found by computing the reduced row echelon form (RREF) [9] of X. In the LIDG method, X is linearly independentized by appropriately removing the detected descriptors having a multicollinearity relationship. Since the constant term is originally included in the regression model, constant terms additionally arising by multiplication are removed tacitly.

### 2.3. Model selection

In sparse modeling [4], the best model that has the highest predictivity is usually selected by the cross-validation procedure [10,11]. For the purpose, we employ the leave-one-out scheme in this study, where 81 sets of data (target and descriptors) are used for the construction of model and the remaining one set of data called j is adopted for estimating the predictivity error [12] as

(3) $$Q_{j}^{2}=1-\frac{{\left(y_{j}-{\hat{y}}_{j}\right)}^{2}}{\sum_{i}{\left(y_{i}-\overset{ˉ}{y}\right)}^{2}},$$

where $y_{i}$, ${\hat{y}}_{i}$, and $\overset{ˉ}{y}$ are true, predicted, and averaged target valuables, respectively. Then, the measure of predictivity for the model selection by the cross-validation is calculated by average as

(4) $$Q^{2}=\frac{1}{N}\sum_{j}Q_{j}^{2}$$

with the total number of data set N (82 in this case). To obtain models as simple as possible, the exhaustive search method [13] for a given number of descriptors is employed.

## 3. Results

**Figure 1.**
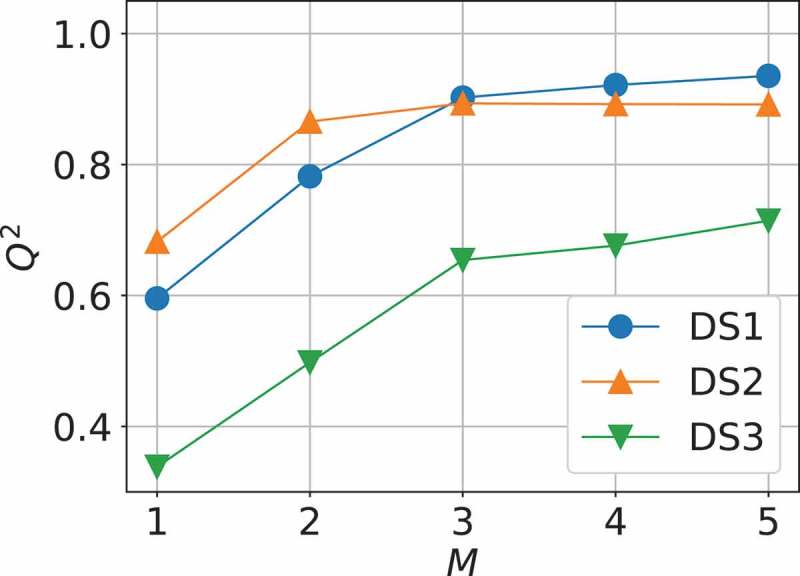
Measure of predictivity $Q^{2}$ for the best models with descriptor space 1 (DS1), 2 (DS2), and 3 (DS3) as a function of the number of descriptors M obtained by the exhaustive search.

Using the procedures described in the previous section with the four kinds of basic descriptors, 86 descriptors are generated up to the second order, called descriptor space 1 (DS1) as listed in Appendix B. Figure 1 shows $Q^{2}$ for the best models by the exhaustive search as a function of the number of descriptors in DS1. That is, when M descriptors are used, linear regressions (ordinary least-squares method) are performed for all the combinations of ${\;}_{p}C_{M}$ descriptors, and $Q^{2}$ of Equation (4) is calculated for each, and then the maximum value of $Q^{2}$ is plotted. Here, p=86 is the total number of descriptors in DS1. Detailed results of model selection are summarized in Appendix C.

**Figure 2.**
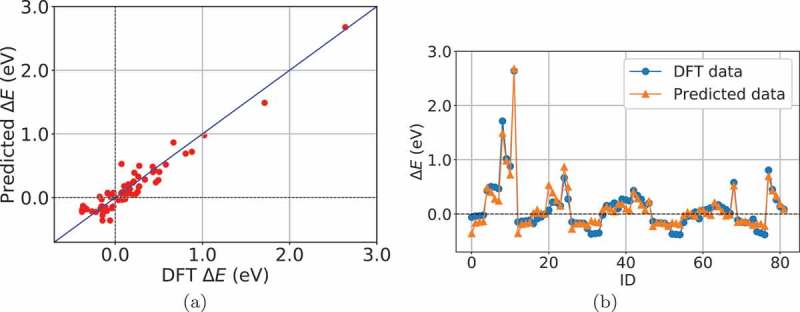
Regression performance of Model 1. (a) predicted and DFT data. (b) predicted and DFT data for each semiconductor. ID corresponds to that in Table A1.

It is seen in Figure 1 that as the number of descriptors M is increased, the predictivity $Q^{2}$ with DS1 is also increased through M=3 and then almost saturated afterward. Therefore, the model with M=3 is appropriately simple with relatively high predictability. This model called Model 1 is given as

(5) $$\begin{array}{rl} & \Delta E=0.59|EN_{A}-EN_{B}|-1.95\frac{|EN_{A}-EN_{B}|}{r_{A}+r_{B}}\\ & \;\;\;\;\;\;\;\;+6.15{\left(\frac{1}{r_{A}+r_{B}}\right)}^{2}-0.75, \end{array}$$

where EN and r are electronegativity and atomic radius, respectively. The regression performance of Model 1 is shown in Figure 2. It is quite interesting that only the electronegativity and atomic radius are included in Model 1 with a simple form, but its physical meaning is not readily understandable.

Electronegativity and atomic radius are known to be empirically correlated as [14,15]

(6) $$EN\propto \frac{1}{r}$$

though they are not so highly collinear that our near-collinearity criteria judge. Note that Pearson’s correlation coefficient is ρ=−0.88. Therefore, atomic radius only and electronegativity only in the basic descriptor set are used on trial to generate 24 high-order descriptors up to fourth order for sparse modeling, called descriptor space 2 (DS2) and 3 (DS3), respectively, as listed in Appendix B. As results, it is found that DS2 gives much better $Q^{2}$ than DS3. For example, $Q^{2}$ for M=5 in DS2 and DS3 is 0.892 and 0.714, respectively.

**Figure 3.**
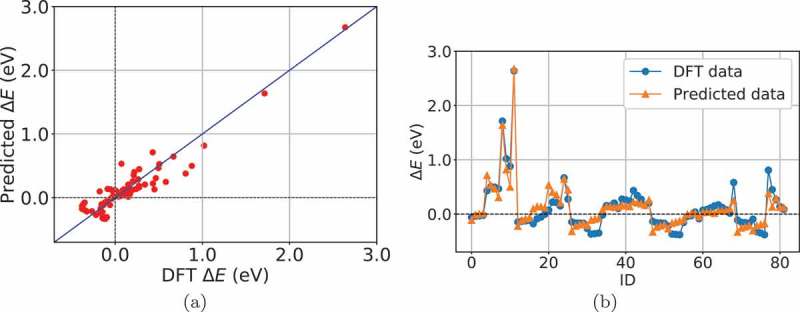
Regression performance of Model 2. (a) predicted and DFT data. (b) predicted and DFT data for each semiconductor. ID corresponds to that in Table A1.

In Figure 1, $Q^{2}$ with DS2 becomes almost constant beyond M=2 and the model with M=2 might be a good one from the viewpoints of predictivity and interpretable sparse modeling, being called Model 2 expressed as

(7) $$\begin{array}{rl} & \Delta E=6.87{\left(\frac{1}{r_{A}+r_{B}}\right)}^{3}-5.02\frac{|r_{A}-r_{B}|}{{\left(r_{A}+r_{B}\right)}^{3}}-0.18\\ & \;\;\;\;\;\;\;\;={\left(\frac{1}{r_{A}+r_{B}}\right)}^{3}\left\{-5.02|r_{A}-r_{B}|+6.87\right\}-0.18 \end{array}$$

and its regression performance is given in Figure 3.

It should be emphasized that Model 2 is a really simple model including atomic-radius descriptors only at high predictivity ($Q^{2}=0.866$). Regression performance of the present models (Model 1 and Model 2) and the previous ones (Model A, Model B, and Model C) is summarized in Table 1 in terms of decision coefficient $R^{2}=1-\sum_{i}(y_{i}-{\hat{y}}_{i}{)}^{2}/\sum_{i}(y_{i}-\overset{ˉ}{y}{)}^{2}$ [16], measure of predictivity $Q^{2}$ (Equation 4) [12], Akaike information criterion AIC $=N\left(\ln(\sum_{i}{\left(y_{i}-{\hat{y}}_{i}\right)}^{2})+\ln(2\pi /N)+1\right)+2M$ [17,18], mean absolute error MAE $=\sum_{i}|y_{i}-{\hat{y}}_{i}|$, and maximum absolute error MaxAE $=\max\left\{\left|y_{i}-{\hat{y}}_{i}\right|,\text{\textbackslash{}break}i=1,2,...,N\right\}$. Model A, Model B, and Model C of the previous work in Table 1 are the best models with descriptors selecting one, two, and three, respectively, from left in the following descriptor list:

(8) $$\frac{IP_{B}-EA_{B}}{r_{pA}^{2}},\frac{|r_{sA}-r_{pB}|}{\exp(r_{sA})},\frac{|r_{pB}-r_{sB}|}{\exp(r_{dA})}.$$

**Table 1.** Regression performance of models obtained in the present and the previous works. M, $R^{2}$, $Q^{2}$, AIC, MAE, and MaxAE are the number of descriptors, decision coefficient [16], measure of predictivity (Equation 4) [12], Akaike information criterion [17,18], mean absolute error, and maximum absolute error, respectively. Models in the previous work are given in the text.

	Present		Previous work [5]		
Criterion	Model 1	Model 2	Model A	Model B	Model C
M	3	2	1	2	3
$R^{2}$	0.913	0.876	0.883	0.929	0.957
$Q^{2}$	0.902	0.866	0.867	0.918	0.946
AIC	−92.4	−65.0	−72.0	−110.6	−149.4
MAE (eV)	0.102	0.118	0.121	0.097	0.071
MaxAE (eV)	0.457	0.460	0.400	0.349	0.301

Note that the values of ionization potential, electron affinity, and electronegativity used in the present study are slightly different from those in the previous work [5]. Because of that, MAE and MaxAE do not perfectly coincide with those listed previously.

## 4. Discussion

Let us consider the possible consequences of Model 2 that is the simplest one among the models constructed in the preceding session. In the cases of elemental materials ($r_{A}=r_{B}$), ΔE becomes positive for r<1.68 Å, preferring zinc-blende (properly diamond) structure. Actually, no elementary materials nor compounds with the same atomic radii greater than 1.68 Å are included in the present octet compounds. For compounds with largely different atomic radii, rock-salt structure with higher coordination than zinc blende is realized. From Equation (7), the borderline between ZB and RS structures, namely ΔE=0, is given as $|r_{A}-r_{B}|=-0.0359(r_{A}+r_{B}{)}^{3}+1.37$, providing a quantitative guideline to classify ZB and RS structures in the present systems. The borderline and the structural classification will be discussed further below in relation to van Arkel-Ketelaar’s triangle of chemical bonding. Approximately, Model 2 shown in Equation (7) tells that the energy difference between ZB and RS structures is linearly scaled to the absolute difference in the atomic radius of the constituent atoms ($\propto |r_{A}-r_{B}|$) and inversely proportional to the cell volume ($\propto (r_{A}+r_{B}{)}^{-3}$). In the octet compounds, the cohesion mechanism is dominated by covalent bonds with additive ionic electrostatic interactions. Covalent bonds originate from the formation of bonding and antibonding states between neighboring orbitals and are roughly proportional to the size of the corresponding hopping integrals. According to the scaling rules in the tight-binding theory [19–21], the hopping integral for neighboring p orbitals is proportional to $R^{-3}$, where R is the interatomic distance. Therefore, it is reasonable to see the inverse proportionality of the cell volume in the energy difference. Chemical trends in the stable structure directly derived from Model 2 are listed in Table 2.

**Table 2.** Relations between atomic radius and stable structure derived from Model 2 (Equation 7). ΔE is defined in Equation 1.

Atomic radius	ΔE	Stable structure
$|r_{A}-r_{B}|$ : large	<0	Rock salt
$r_{A}+r_{B}$ : small		
and	>0	Zinc blende
$|r_{A}-r_{B}|$ : small		

Empirically, electronegativity is well known to be related to chemical bonding in compounds [22] and has an inverse relation to the atomic radius, as shown in Equation (6). With this relation, the trends with respect to the atomic radius listed in Table 2 can be converted to trends with respect to electronegativity given in Table 3. This result is consistent with our knowledge of the stable structure in semiconductors such that covalent (ionic) compounds tend to possess zinc-blende (rock-salt) crystal structure [23]. Nevertheless, it is quite interesting to able to model the energy difference ΔE quantitatively better with atomic radius than with electronegativity, as mentioned in the preceding section.

**Table 3.** Relations between electronegativity, stable structure, and chemical bond, derived from Table 2 and Equation 6.

Electronegativity	ΔE	Stable structure	Chemical bond
$|EN_{A}-EN_{B}|$ : large	<0	Rock salt	Ionic
$EN_{A}+EN_{B}$ : large			
and	>0	Zinc blende	Covalent
$|EN_{A}-EN_{B}|$ : small			

van Arkel-Ketelaar’s triable is a map for displaying chemical bonding of compounds [24–26]. Metallic, ionic, and covalent bonding are represented in a two-dimensional (2D) map with the axes of mean and difference of electronegativity of the constituent atoms in the latest version [27,28]. Following van Arkel-Ketelaar’s triangle, the total energy difference given by Equation (7) is plotted in a 2D map of the sum and difference of atomic radius as shown in Figure 4. Figure 4 precisely reproduces the stable crystal structure, either zinc-blende or rock-salt and covalent or ionic bonding via relation between structure and chemical bonding. Note that the models constructed by regression include no information about chemical bonding characteristics beyond the training data. As a matter of fact, Model 2 can not represent metallic systems, that may correspond to an empty region in the present triangle shown in Figure 4.

**Figure 4.**
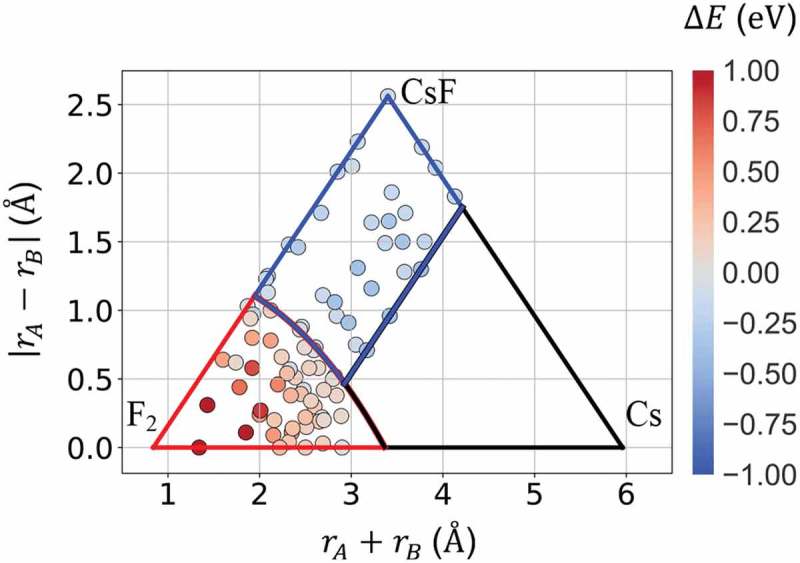
Total energy difference map in a triable of the sum and difference of atomic radius of the constituent atoms given by Model 2 (Equation 7). Red-colored (blue-colored) dots form an area where zinc-blende (rock-salt) structure is stable and covalent (ionic) bonding is realized. An area with no dots corresponds to the region where training data are not included, possibly indicating a metallic bonding region.

## 5. Conclusions

A simple model quantifying energy difference between zinc-blende and rock-salt structure in octet elemental and binary semiconductors is obtained with only the information of atomic radius of constituent atoms, leading to a 2D map of chemical bonding represented in terms of the sum and difference of atomic radius. It is found that our descriptor-generation method including symmetrization for permutation, multiplication operation to higher order, and removal of collinearity problems is crucial to construct such a sparse model in addition to the exhaustive search. That is, since we use only symmetrized descriptors as initial descriptors, it is guaranteed that a correct model can be obtained at least in terms of symmetry. In addition, since inappropriate descriptors that do not satisfy symmetry are not included, the number of descriptor candidates can be reduced. The above two are the effects of descriptor symmetrization. On the other hand, the model obtained in the previous study does not satisfy the symmetry due to the permutation of A and B elements. Therefore, no matter how high the prediction accuracy, it can be said that this is a physically inappropriate model at least in symmetry. One would also like to mention the effect of removing multicollinearity. For example, if there is multicollinearity, such as $x_{i}=ax_{j}+bx_{k}$, in descriptor matrix, the estimation and prediction accuracies do not change regardless of which one of $x_{i}$, $x_{j}$ and $x_{k}$ is deleted from the descriptor matrix. Therefore, it cannot be decided from statistics whether $x_{i}$, $x_{j}$, or $x_{k}$ should be removed. In our LIDG method, however, since the multicollinearity has been detected prior to regression, one can introduce the simplicity of descriptors in the descriptor selection process and employ two descriptors with a simpler form between $x_{i}$, $x_{j}$, and $x_{k}$. Therefore, the obtained model is the simplest model among the models that give the same prediction accuracy. This is the advantage of the LIDG method in the detection and removal of multicollinearity.

## Appendix A. Regression data

Target and descriptor data used in the present study are listed in Tables A1 and A2, respectively.

**Table A1.** Target data for regression taken from Ghiringhelli et al. [5]. ΔE is total energy difference (in eV/atom) between zinc-blende and rock-salt structures ΔE=E(RS)−E(ZB) of 82 elementary and binary systems AB.

ID	A	B	ΔE	ID	A	B	ΔE	ID	A	B	ΔE
0	Li	F	−0.059	28	Si	Si	0.275	56	Rb	I	−0.169
1	Li	Cl	−0.038	29	Si	Ge	0.264	57	Sr	O	−0.221
2	Li	Br	−0.033	30	Si	Sn	0.136	58	Sr	S	−0.369
3	Li	I	−0.022	31	K	F	−0.146	59	Sr	Se	−0.375
4	Be	O	0.430	32	K	Cl	−0.165	60	Sr	Te	−0.381
5	Be	S	0.506	33	K	Br	−0.166	61	Ag	F	−0.156
6	Be	Se	0.495	34	K	I	−0.168	62	Ag	Cl	−0.044
7	Be	Te	0.466	35	Ca	O	−0.266	63	Ag	Br	−0.030
8	B	N	1.713	36	Ca	S	−0.369	64	Ag	I	0.037
9	B	P	1.020	37	Ca	Se	−0.361	65	Cd	O	−0.087
10	B	As	0.879	38	Ca	Te	−0.350	66	Cd	S	0.070
11	B	Sb	0.581	39	Cu	F	−0.019	67	Cd	Se	0.083
12	C	C	2.638	40	Cu	Cl	0.156	68	Cd	Te	0.113
13	C	Si	0.668	41	Cu	Br	0.152	69	In	N	0.150
14	C	Ge	0.808	42	Cu	I	0.203	70	In	P	0.170
15	C	Sn	0.450	43	Zn	O	0.102	71	In	As	0.122
16	Na	F	−0.146	44	Zn	S	0.275	72	In	Sb	0.080
17	Na	Cl	−0.133	45	Zn	Se	0.259	73	Sn	Sn	0.016
18	Na	Br	−0.127	46	Zn	Te	0.241	74	Cs	F	−0.112
19	Na	I	−0.115	47	Ga	N	0.433	75	Cs	Cl	−0.152
20	Mg	O	−0.178	48	Ga	P	0.341	76	Cs	Br	−0.158
21	Mg	S	−0.087	49	Ga	As	0.271	77	Cs	I	−0.165
22	Mg	Se	−0.055	50	Ga	Sb	0.158	78	Ba	O	−0.095
23	Mg	Te	−0.005	51	Ge	Ge	0.202	79	Ba	S	−0.326
24	Al	N	0.072	52	Ge	Sn	0.087	80	Ba	Se	−0.350
25	Al	P	0.219	53	Rb	F	−0.136	81	Ba	Te	−0.381
26	Al	As	0.212	54	Rb	Cl	−0.161				
27	Al	Sb	0.150	55	Rb	Br	−0.164

**Table A2.** Basic descriptors. r, IP, EA, and EN are atomic radius, ionization potential, electron affinity, and electronegativity, respectively. $r_{s}$, $r_{p}$, and $r_{d}$ are the radius at maximum probability amplitude of s, p, and d orbitals, respectively.

Atom	$r^{a}$ (Å)	$IP^{b}$ (eV)	$EA^{c}$ (eV)	$EN^{d}$	$r_{s}^{e}$ (Å)	$r_{p}^{e}$ (Å)	$r_{d}^{e}$ (Å)
Li	1.67	5.392	0.6180	0.98	1.652	1.995	6.930
Be	1.12	9.322	−0.5000	1.57	1.078	1.211	2.877
B	0.87	8.298	0.2770	2.04	0.805	0.826	1.946
C	0.67	11.260	1.2629	2.55	0.644	0.630	1.631
N	0.56	14.534	−0.0700	3.04	0.539	0.511	1.540
O	0.48	13.618	1.4611	3.44	0.462	0.427	2.219
F	0.42	17.422	3.3990	3.98	0.406	0.371	1.428
Na	1.90	5.139	0.5479	0.93	1.715	2.597	6.566
Mg	1.45	7.646	−0.4000	1.31	1.330	1.897	3.171
Al	1.18	5.986	0.4410	1.61	1.092	1.393	1.939
Si	1.11	8.151	1.3850	1.90	0.938	1.134	1.890
P	0.98	10.486	0.7465	2.19	0.826	0.966	1.771
S	0.88	10.360	2.0771	2.58	0.742	0.847	2.366
Cl	0.79	12.967	3.6170	3.16	0.679	0.756	1.666
K	2.43	4.341	0.5015	0.82	2.128	2.443	1.785
Ca	1.94	6.113	−0.3000	1.00	1.757	2.324	0.679
Cu	1.45	7.726	1.2280	1.90	1.197	1.680	2.576
Zn	1.42	9.394	−0.6000	1.65	1.099	1.547	2.254
Ga	1.36	5.999	0.3000	1.81	0.994	1.330	2.163
Ge	1.25	7.899	1.2000	2.01	0.917	1.162	2.373
As	1.14	9.810	0.8100	2.18	0.847	1.043	2.023
Se	1.03	9.752	2.0207	2.55	0.798	0.952	2.177
Br	0.94	11.814	3.3650	2.96	0.749	0.882	1.869
Rb	2.65	4.177	0.4859	0.82	2.240	3.199	1.960
Sr	2.19	5.695	−0.3000	0.95	1.911	2.548	1.204
Ag	1.65	7.576	1.3020	1.93	1.316	1.883	2.968
Cd	1.61	8.993	−0.7000	1.69	1.232	1.736	2.604
In	1.56	5.786	0.3000	1.78	1.134	1.498	3.108
Sn	1.45	7.344	1.2000	1.96	1.057	1.344	2.030
Sb	1.33	8.641	1.0700	2.05	1.001	1.232	2.065
Te	1.23	9.009	1.9708	2.10	0.945	1.141	1.827
I	1.15	10.451	3.0591	2.66	0.896	1.071	1.722
Cs	2.98	3.894	0.4716	0.79	2.464	3.164	1.974
Ba	2.53	5.212	−0.3000	0.89	2.149	2.632	1.351

^a^Ref. [14]:,^b^Ref. [29]:,^c^Ref. [30]:,^d^Ref. [15]:,^e^Ref. [5].

## Appendix B. Descriptor space

Descriptors included in descriptor space 1 (DS1), 2 (DS2), and 3 (DS3) are listed in Tables B1-B3, respectively.

**Table B1.** Descriptor space 1 (DS1): 86 descriptors up to second order of atomic radius, ionization potential, electron affinity, and electronegativity.

Order	Descriptor
1	$r_{A}+r_{B}$, $IP_{A}+IP_{B}$, $EA_{A}+EA_{B}$, $EN_{A}+EN_{B}$
	$|r_{A}-r_{B}|$, $|IP_{A}-IP_{B}|$, $|EA_{A}-EA_{B}|$, $|EN_{A}-EN_{B}|$
	$\frac{1}{r_{A}+r_{B}}$, $\frac{1}{IP_{A}+IP_{B}}$, $\frac{1}{EA_{A}+EA_{B}}$, $\frac{1}{EN_{A}+EN_{B}}$
2	${\left(r_{A}+r_{B}\right)}^{2}$, $\left(r_{A}+r_{B}\right)\left(IP_{A}+IP_{B}\right)$, $\left(r_{A}+r_{B}\right)\left(EA_{A}+EA_{B}\right)$
	$\left(r_{A}+r_{B}\right)\left(EN_{A}+EN_{B}\right)$, $\left(r_{A}+r_{B}\right)|r_{A}-r_{B}|$, $\left(r_{A}+r_{B}\right)|IP_{A}-IP_{B}|$
	$\left(r_{A}+r_{B}\right)|EA_{A}-EA_{B}|$, $\left(r_{A}+r_{B}\right)|EN_{A}-EN_{B}|$, $\frac{r_{A}+r_{B}}{IP_{A}+IP_{B}}$
	$\frac{r_{A}+r_{B}}{EA_{A}+EA_{B}}$, $\frac{r_{A}+r_{B}}{EN_{A}+EN_{B}}$, ${\left(IP_{A}+IP_{B}\right)}^{2}$, $\left(IP_{A}+IP_{B}\right)\left(EA_{A}+EA_{B}\right)$
	$\left(IP_{A}+IP_{B}\right)\left(EN_{A}+EN_{B}\right)$, $\left(IP_{A}+IP_{B}\right)|r_{A}-r_{B}|$
	$\left(IP_{A}+IP_{B}\right)|IP_{A}-IP_{B}|$, $\left(IP_{A}+IP_{B}\right)|EA_{A}-EA_{B}|$
	$\left(IP_{A}+IP_{B}\right)|EN_{A}-EN_{B}|$, $\frac{IP_{A}+IP_{B}}{r_{A}+r_{B}}$, $\frac{IP_{A}+IP_{B}}{EA_{A}+EA_{B}}$, $\frac{IP_{A}+IP_{B}}{EN_{A}+EN_{B}}$
	${\left(EA_{A}+EA_{B}\right)}^{2}$, $\left(EA_{A}+EA_{B}\right)\left(EN_{A}+EN_{B}\right)$, $\left(EA_{A}+EA_{B}\right)|r_{A}-r_{B}|$
	$\left(EA_{A}+EA_{B}\right)|IP_{A}-IP_{B}|$, $\left(EA_{A}+EA_{B}\right)|EA_{A}-EA_{B}|$
	$\left(EA_{A}+EA_{B}\right)|EN_{A}-EN_{B}|$, $\frac{EA_{A}+EA_{B}}{r_{A}+r_{B}}$, $\frac{EA_{A}+EA_{B}}{IP_{A}+IP_{B}}$, $\frac{EA_{A}+EA_{B}}{EN_{A}+EN_{B}}$
	${\left(EN_{A}+EN_{B}\right)}^{2}$, $\left(EN_{A}+EN_{B}\right)|r_{A}-r_{B}|$, $\left(EN_{A}+EN_{B}\right)|IP_{A}-IP_{B}|$
	$\left(EN_{A}+EN_{B}\right)|EA_{A}-EA_{B}|$, $\left(EN_{A}+EN_{B}\right)|EN_{A}-EN_{B}|$
	$\frac{EN_{A}+EN_{B}}{r_{A}+r_{B}}$, $\frac{EN_{A}+EN_{B}}{IP_{A}+IP_{B}}$, $\frac{EN_{A}+EN_{B}}{EA_{A}+EA_{B}}$, $|r_{A}-r_{B}{|}^{2}$, $|r_{A}-r_{B}||IP_{A}-IP_{B}|$
	$|r_{A}-r_{B}||EA_{A}-EA_{B}|$, $|r_{A}-r_{B}||EN_{A}-EN_{B}|$, $\frac{|r_{A}-r_{B}|}{r_{A}+r_{B}}$, $\frac{|r_{A}-r_{B}|}{IP_{A}+IP_{B}}$
	$\frac{|r_{A}-r_{B}|}{EA_{A}+EA_{B}}$, $\frac{|r_{A}-r_{B}|}{EN_{A}+EN_{B}}$, $|IP_{A}-IP_{B}{|}^{2}$, $|IP_{A}-IP_{B}||EA_{A}-EA_{B}|$
	$|IP_{A}-IP_{B}||EN_{A}-EN_{B}|$, $\frac{|IP_{A}-IP_{B}|}{r_{A}+r_{B}}$, $\frac{|IP_{A}-IP_{B}|}{IP_{A}+IP_{B}}$, $\frac{|IP_{A}-IP_{B}|}{EA_{A}+EA_{B}}$, $\frac{|IP_{A}-IP_{B}|}{EN_{A}+EN_{B}}$
	$|EA_{A}-EA_{B}{|}^{2}$, $|EA_{A}-EA_{B}||EN_{A}-EN_{B}|$, $\frac{|EA_{A}-EA_{B}|}{r_{A}+r_{B}}$, $\frac{|EA_{A}-EA_{B}|}{IP_{A}+IP_{B}}$
	$\frac{|EA_{A}-EA_{B}|}{EA_{A}+EA_{B}}$, $\frac{|EA_{A}-EA_{B}|}{EN_{A}+EN_{B}}$, $|EN_{A}-EN_{B}{|}^{2}$, $\frac{|EN_{A}-EN_{B}|}{r_{A}+r_{B}}$, $\frac{|EN_{A}-EN_{B}|}{IP_{A}+IP_{B}}$
	$\frac{|EN_{A}-EN_{B}|}{EA_{A}+EA_{B}}$, $\frac{|EN_{A}-EN_{B}|}{EN_{A}+EN_{B}}$, ${\left(\frac{1}{r_{A}+r_{B}}\right)}^{2}$, $\frac{1}{\left(r_{A}+r_{B}\right)\left(IP_{A}+IP_{B}\right)}$
	$\frac{1}{\left(r_{A}+r_{B}\right)\left(EA_{A}+EA_{B}\right)}$, $\frac{1}{\left(r_{A}+r_{B}\right)\left(EN_{A}+EN_{B}\right)}$, ${\left(\frac{1}{IP_{A}+IP_{B}}\right)}^{2}$
	$\frac{1}{\left(IP_{A}+IP_{B}\right)\left(EA_{A}+EA_{B}\right)}$, $\frac{1}{\left(IP_{A}+IP_{B}\right)\left(EN_{A}+EN_{B}\right)}$, ${\left(\frac{1}{EA_{A}+EA_{B}}\right)}^{2}$
	$\frac{1}{\left(EA_{A}+EA_{B}\right)\left(EN_{A}+EN_{B}\right)}$, ${\left(\frac{1}{EN_{A}+EN_{B}}\right)}^{2}$

**Table B2.** Descriptor space 2 (DS2): 24 descriptors up to fourth order of atomic radius.

Order	Descriptors
1	$r_{A}+r_{B}$, $|r_{A}-r_{B}|$, $\frac{1}{r_{A}+r_{B}}$
2	${\left(r_{A}+r_{B}\right)}^{2}$, $\left(r_{A}+r_{B}\right)|r_{A}-r_{B}|$, $|r_{A}-r_{B}{|}^{2}$, $\frac{|r_{A}-r_{B}|}{r_{A}+r_{B}}$
	${\left(\frac{1}{r_{A}+r_{B}}\right)}^{2}$
3	${\left(r_{A}+r_{B}\right)}^{3}$, ${\left(r_{A}+r_{B}\right)}^{2}|r_{A}-r_{B}|$, $\left(r_{A}+r_{B}\right)|r_{A}-r_{B}{|}^{2}$
	$|r_{A}-r_{B}{|}^{3}$, $\frac{|r_{A}-r_{B}{|}^{2}}{r_{A}+r_{B}}$, $\frac{|r_{A}-r_{B}|}{{\left(r_{A}+r_{B}\right)}^{2}}$, ${\left(\frac{1}{r_{A}+r_{B}}\right)}^{3}$
4	${\left(r_{A}+r_{B}\right)}^{4}$, ${\left(r_{A}+r_{B}\right)}^{3}|r_{A}-r_{B}|$, ${\left(r_{A}+r_{B}\right)}^{2}|r_{A}-r_{B}{|}^{2}$
	$\left(r_{A}+r_{B}\right)|r_{A}-r_{B}{|}^{3}$, $|r_{A}-r_{B}{|}^{4}$, $\frac{|r_{A}-r_{B}{|}^{3}}{\left(r_{A}+r_{B}\right)}$, $\frac{|r_{A}-r_{B}{|}^{2}}{{\left(r_{A}+r_{B}\right)}^{2}}$
	$\frac{|r_{A}-r_{B}|}{{\left(r_{A}+r_{B}\right)}^{3}}$, ${\left(\frac{1}{r_{A}+r_{B}}\right)}^{4}$

**Table B3.** Descriptor space 3 (DS3): 24 descriptors up to fourth order of electronegativity.

Order	Descriptors
1	$EN_{A}+EN_{B}$, $|EN_{A}-EN_{B}|$, $\frac{1}{EN_{A}+EN_{B}}$
2	${\left(EN_{A}+EN_{B}\right)}^{2}$, $\left(EN_{A}+EN_{B}\right)|EN_{A}-EN_{B}|$, $|EN_{A}-EN_{B}{|}^{2}$
	$\frac{|EN_{A}-EN_{B}|}{EN_{A}+EN_{B}}$, ${\left(\frac{1}{EN_{A}+EN_{B}}\right)}^{2}$
3	${\left(EN_{A}+EN_{B}\right)}^{3}$, ${\left(EN_{A}+EN_{B}\right)}^{2}|EN_{A}-EN_{B}|$
	$\left(EN_{A}+EN_{B}\right)|EN_{A}-EN_{B}{|}^{2}$, $|EN_{A}-EN_{B}{|}^{3}$
	$\frac{|EN_{A}-EN_{B}{|}^{2}}{EN_{A}+EN_{B}}$, $\frac{|EN_{A}-EN_{B}|}{{\left(EN_{A}+EN_{B}\right)}^{2}}$, ${\left(\frac{1}{EN_{A}+EN_{B}}\right)}^{3}$
4	${\left(EN_{A}+EN_{B}\right)}^{4}$, ${\left(EN_{A}+EN_{B}\right)}^{3}|EN_{A}-EN_{B}|$
	${\left(EN_{A}+EN_{B}\right)}^{2}|EN_{A}-EN_{B}{|}^{2}$, $\left(EN_{A}+EN_{B}\right)|EN_{A}-EN_{B}{|}^{3}$
	$|EN_{A}-EN_{B}{|}^{4}$, $\frac{|EN_{A}-EN_{B}{|}^{3}}{\left(EN_{A}+EN_{B}\right)}$, $\frac{|EN_{A}-EN_{B}{|}^{2}}{{\left(EN_{A}+EN_{B}\right)}^{2}}$, $\frac{|EN_{A}-EN_{B}|}{{\left(EN_{A}+EN_{B}\right)}^{3}}$
	${\left(\frac{1}{EN_{A}+EN_{B}}\right)}^{4}$

## Appendix C. Results of descriptor selection by exhaustive search

The results of descriptor (model) selection by exhaustive search are summarized in Tables C1–C3, and for descriptor space 1 (DS1), 2 (DS2), and 3 (DS3), respectively. Not only the best but also the second and third best models are listed for each M.

**Table C1.** Results of model selection in descriptor space 1 (DS1) by exhaustive search. Models are ranked by $Q^{2}$ score and listed only top 3.

M	Ranking	$R^{2}$	$Q^{2}$	ΔE
1	1	0.658	0.595	4.12${\left(\frac{1}{r_{A}+r_{B}}\right)}^{2}$ −0.60
	2	0.592	0.522	3.56$\frac{1}{r_{A}+r_{B}}$ −1.33
	3	0.546	0.481	86.63$\frac{1}{r_{A}+r_{B}}$$\frac{1}{IP_{A}+IP_{B}}$ −1.82
2	1	0.823	0.782	−0.53$\frac{|EN_{A}-EN_{B}|}{r_{A}+r_{B}}$ + 4.25${\left(\frac{1}{r_{A}+r_{B}}\right)}^{2}$ 0.36
	2	0.771	0.728	−0.17$\frac{IP_{A}+IP_{B}}{r_{A}+r_{B}}$ + 9.11${\left(\frac{1}{r_{A}+r_{B}}\right)}^{2}$ −0.20
	3	0.767	0.716	−0.11$\frac{|IP_{A}-IP_{B}|}{r_{A}+r_{B}}$ + 4.42${\left(\frac{1}{r_{A}+r_{B}}\right)}^{2}$ −0.46
3	1	0.913	0.902	0.59$|EN_{A}-EN_{B}|$ −1.95$\frac{|EN_{A}-EN_{B}|}{r_{A}+r_{B}}$
				+ 6.15${\left(\frac{1}{r_{A}+r_{B}}\right)}^{2}$ −0.75
	2	0.911	0.899	0.12$|EA_{A}-EA_{B}|$$|EN_{A}-EN_{B}|$ −1.26$\frac{|EN_{A}-EN_{B}|}{r_{A}+r_{B}}$
				+ 5.77${\left(\frac{1}{r_{A}+r_{B}}\right)}^{2}$ −0.60
	3	0.904	0.892	0.10$\left(r_{A}+r_{B}\right)$$|EN_{A}-EN_{B}|$ −1.13$\frac{|EN_{A}-EN_{B}|}{r_{A}+r_{B}}$
				+ 5.89${\left(\frac{1}{r_{A}+r_{B}}\right)}^{2}$ −0.70
4	1	0.934	0.921	1.86$\frac{|EA_{A}-EA_{B}|}{IP_{A}+IP_{B}}$+ 0.13$|EN_{A}-EN_{B}{|}^{2}$
				−1.52$\frac{|EN_{A}-EN_{B}|}{r_{A}+r_{B}}$ + 6.14${\left(\frac{1}{r_{A}+r_{B}}\right)}^{2}$ −0.68
	2	0.933	0.921	0.04$\left(r_{A}+r_{B}\right)$$|EA_{A}-EA_{B}|$ + 0.11$|EN_{A}-EN_{B}{|}^{2}$
				−1.42$\frac{|EN_{A}-EN_{B}|}{r_{A}+r_{B}}$ + 6.09${\left(\frac{1}{r_{A}+r_{B}}\right)}^{2}$ −0.67
	3	0.930	0.920	0.13$\left(r_{A}+r_{B}\right)$$|EN_{A}-EN_{B}|$ −0.06$\left(IP_{A}+IP_{B}\right)$$|EN_{A}-EN_{B}|$
				+ 0.22$\frac{IP_{A}+IP_{B}}{r_{A}+r_{B}}$ + 0.23$|EN_{A}-EN_{B}{|}^{2}$ −1.00
5	1	0.945	0.936	0.22$\left(r_{A}+r_{B}\right)$$|EN_{A}-EN_{B}|$ −0.07$\left(IP_{A}+IP_{B}\right)$$|EN_{A}-EN_{B}|$
				+ 0.22$\frac{IP_{A}+IP_{B}}{r_{A}+r_{B}}$ −1.06$\frac{|r_{A}-r_{B}|}{EN_{A}+EN_{B}}$
				+ 0.21$|EN_{A}-EN_{B}{|}^{2}$ −0.10
	2	0.946	0.936	−0.01$\left(IP_{A}+IP_{B}\right)$$\left(EA_{A}+EA_{B}\right)$
				+ 0.05$\left(EA_{A}+EA_{B}\right)$$|EA_{A}-EA_{B}|$ + 0.11$|EN_{A}-EN_{B}{|}^{2}$
				−1.50$\frac{|EN_{A}-EN_{B}|}{r_{A}+r_{B}}$ + 6.16${\left(\frac{1}{r_{A}+r_{B}}\right)}^{2}$ −0.53
	3	0.944	0.935	0.22$\left(r_{A}+r_{B}\right)$$|EN_{A}-EN_{B}|$ −0.07$\left(IP_{A}+IP_{B}\right)$$|EN_{A}-EN_{B}|$
				+ 0.22$\frac{IP_{A}+IP_{B}}{r_{A}+r_{B}}$ −4.55$\frac{|r_{A}-r_{B}|}{IP_{A}+IP_{B}}$
				+ 0.21$|EN_{A}-EN_{B}{|}^{2}$ −1.01

**Table C2.** Results of model selection in descriptor space 2 (DS2) by exhaustive search. Models are ranked by $Q^{2}$ score and listed only top 3.

M	Ranking	$R^{2}$	$Q^{2}$	ΔE
1	1	0.711	0.682	8.08${\left(\frac{1}{r_{A}+r_{B}}\right)}^{4}$ −0.19
	2	0.698	0.652	5.70${\left(\frac{1}{r_{A}+r_{B}}\right)}^{3}$ −0.34
	3	0.658	0.595	4.12${\left(\frac{1}{r_{A}+r_{B}}\right)}^{2}$ −0.60
2	1	0.876	0.866	6.87${\left(\frac{1}{r_{A}+r_{B}}\right)}^{3}$ −5.02$\frac{|r_{A}-r_{B}|}{{\left(r_{A}+r_{B}\right)}^{3}}$ −0.18
	2	0.863	0.844	5.16${\left(\frac{1}{r_{A}+r_{B}}\right)}^{2}$ −5.49$\frac{|r_{A}-r_{B}|}{{\left(r_{A}+r_{B}\right)}^{3}}$ −0.51
	3	0.844	0.833	−2.01$\frac{|r_{A}-r_{B}|}{{\left(r_{A}+r_{B}\right)}^{2}}$+ 8.43${\left(\frac{1}{r_{A}+r_{B}}\right)}^{4}$+ 0.03
3	1	0.903	0.893	0.08$|r_{A}-r_{B}{|}^{2}$+ 6.03${\left(\frac{1}{r_{A}+r_{B}}\right)}^{2}$ −7.03$\frac{|r_{A}-r_{B}|}{{\left(r_{A}+r_{B}\right)}^{3}}$ −0.68
	2	0.902	0.893	5.97${\left(\frac{1}{r_{A}+r_{B}}\right)}^{2}$+ 0.02$\left(r_{A}+r_{B}\right)$$|r_{A}-r_{B}{|}^{2}$
				−6.61$\frac{|r_{A}-r_{B}|}{{\left(r_{A}+r_{B}\right)}^{3}}$ −0.67
	3	0.902	0.892	5.85${\left(\frac{1}{r_{A}+r_{B}}\right)}^{2}$ + 0.04$|r_{A}-r_{B}{|}^{3}$ −6.58$\frac{|r_{A}-r_{B}|}{{\left(r_{A}+r_{B}\right)}^{3}}$ −0.64
4	1	0.903	0.892	0.02$\left(r_{A}+r_{B}\right)$$|r_{A}-r_{B}|$ + 6.04${\left(\frac{1}{r_{A}+r_{B}}\right)}^{2}$ + 0.08$\frac{|r_{A}-r_{B}{|}^{3}}{r_{A}+r_{B}}$
				−7.01$\frac{|r_{A}-r_{B}|}{{\left(r_{A}+r_{B}\right)}^{3}}$ −0.69
	2	0.903	0.892	5.99${\left(\frac{1}{r_{A}+r_{B}}\right)}^{2}$ + 0.01$(r_{A}+r_{B}{)}^{2}$$|r_{A}-r_{B}|$ + 0.09$\frac{|r_{A}-r_{B}{|}^{3}}{r_{A}+r_{B}}$
				−6.84$\frac{|r_{A}-r_{B}|}{{\left(r_{A}+r_{B}\right)}^{3}}$ −0.67
	3	0.903	0.892	0.07$|r_{A}-r_{B}{|}^{2}$ + 6.00${\left(\frac{1}{r_{A}+r_{B}}\right)}^{2}$ + 0.03$\frac{|r_{A}-r_{B}{|}^{3}}{r_{A}+r_{B}}$
				−7.01$\frac{|r_{A}-r_{B}|}{{\left(r_{A}+r_{B}\right)}^{3}}$ −0.67
5	1	0.905	0.892	−0.05$(r_{A}+r_{B}{)}^{3}$ + 0.03$\left(r_{A}+r_{B}\right)$$|r_{A}-r_{B}{|}^{2}$
				+ 5.83${\left(\frac{1}{r_{A}+r_{B}}\right)}^{3}$ + 0.01$(r_{A}+r_{B}{)}^{4}$ −6.65$\frac{|r_{A}-r_{B}|}{{\left(r_{A}+r_{B}\right)}^{3}}$+ 0.34
	2	0.904	0.892	−0.12$|r_{A}-r_{B}{|}^{2}$ + 5.88${\left(\frac{1}{r_{A}+r_{B}}\right)}^{2}$ + 0.18$|r_{A}-r_{B}{|}^{3}$
				−0.04$|r_{A}-r_{B}{|}^{4}$ −6.56$\frac{|r_{A}-r_{B}|}{{\left(r_{A}+r_{B}\right)}^{3}}$ −0.64
	3	0.905	0.891	−0.12$(r_{A}+r_{B}{)}^{2}$ + 0.03$\left(r_{A}+r_{B}\right)$$|r_{A}-r_{B}{|}^{2}$ + 5.60${\left(\frac{1}{r_{A}+r_{B}}\right)}^{3}$
				+ 0.003$(r_{A}+r_{B}{)}^{4}$ −6.69$\frac{|r_{A}-r_{B}|}{{\left(r_{A}+r_{B}\right)}^{3}}$ + 0.54

**Table C3.** Results of model selection in descriptor space 3 (DS3) by exhaustive search. Models are ranked by $Q^{2}$ score and listed only top 3.

M	Ranking	$R^{2}$	$Q^{2}$	ΔE
1	1	0.375	0.338	−19.07$\frac{|EN_{A}-EN_{B}|}{{\left(EN_{A}+EN_{B}\right)}^{3}}$ + 0.47
	2	0.375	0.336	−5.25$\frac{|EN_{A}-EN_{B}|}{{\left(EN_{A}+EN_{B}\right)}^{2}}$ + 0.51
	3	0.336	0.294	−1.27$\frac{|EN_{A}-EN_{B}|}{EN_{A}+EN_{B}}$ + 0.50
2	1	0.572	0.498	0.72$\left(EN_{A}+EN_{B}\right)$ −0.004$(EN_{A}+EN_{B}{)}^{3}$$|EN_{A}-EN_{B}|$
				−2.47
	2	0.565	0.482	0.08$(EN_{A}+EN_{B}{)}^{2}$ −0.004$(EN_{A}+EN_{B}{)}^{3}$$|EN_{A}-EN_{B}|$
				−0.94
	3	0.546	0.478	−11.54$\frac{1}{EN_{A}+EN_{B}}$ −0.004$(EN_{A}+EN_{B}{)}^{3}$$|EN_{A}-EN_{B}|$
				+ 3.31
3	1	0.708	0.654	0.07$\left(EN_{A}+EN_{B}\right)$$|EN_{A}-EN_{B}{|}^{2}$ + 0.004$(EN_{A}+EN_{B}{)}^{4}$
				−0.01$(EN_{A}+EN_{B}{)}^{3}$$|EN_{A}-EN_{B}|$ −0.44
	2	0.708	0.646	0.02$(EN_{A}+EN_{B}{)}^{3}$ + 0.05$\left(EN_{A}+EN_{B}\right)$$|EN_{A}-EN_{B}{|}^{2}$
				−0.01$(EN_{A}+EN_{B}{)}^{3}$$|EN_{A}-EN_{B}|$ −0.85
	3	0.697	0.630	0.02$(EN_{A}+EN_{B}{)}^{3}$ + 0.06$|EN_{A}-EN_{B}{|}^{3}$
				−0.01$(EN_{A}+EN_{B}{)}^{3}$$|EN_{A}-EN_{B}|$ −0.72
4	1	0.728	0.676	0.05$(EN_{A}+EN_{B}{)}^{2}$$|EN_{A}-EN_{B}|$ + 0.004$(EN_{A}+EN_{B}{)}^{4}$
				−0.02$(EN_{A}+EN_{B}{)}^{3}$$|EN_{A}-EN_{B}|$
				+ 0.01$(EN_{A}+EN_{B}{)}^{2}$$|EN_{A}-EN_{B}{|}^{2}$ −0.60
	2	0.725	0.673	0.11$\left(EN_{A}+EN_{B}\right)$$|EN_{A}-EN_{B}|$ + 0.004$(EN_{A}+EN_{B}{)}^{4}$
				−0.02$(EN_{A}+EN_{B}{)}^{3}$$|EN_{A}-EN_{B}|$
				+ 0.01$\left(EN_{A}+EN_{B}^{2}\right)$$|EN_{A}-EN_{B}{|}^{2}$ −0.61
	3	0.718	0.662	0.32$|EN_{A}-EN_{B}|$ + 0.004$(EN_{A}+EN_{B}{)}^{4}$
				−0.02$(EN_{A}+EN_{B}{)}^{3}$$|EN_{A}-EN_{B}|$
				+ 0.01$(EN_{A}+EN_{B}{)}^{2}$$|EN_{A}-EN_{B}{|}^{2}$ −0.60
5	1	0.754	0.714	1.20$\left(EN_{A}+EN_{B}\right)$$|EN_{A}-EN_{B}|$
				−0.32$(EN_{A}+EN_{B}{)}^{2}$$|EN_{A}-EN_{B}|$ + 0.005$(EN_{A}+EN_{B}{)}^{4}$
				+ 0.03$(EN_{A}+EN_{B}{)}^{2}$$|EN_{A}-EN_{B}{|}^{2}$
				−6.10${\left(\frac{|EN_{A}-EN_{B}|}{EN_{A}+EN_{B}}\right)}^{2}$ −0.91
	2	0.752	0.709	1.08$\left(EN_{A}+EN_{B}\right)$$|EN_{A}-EN_{B}|$
				−0.29$(EN_{A}+EN_{B}{)}^{2}$$|EN_{A}-EN_{B}|$
				+ 0.19$\left(EN_{A}+EN_{B}\right)$$|EN_{A}-EN_{B}{|}^{2}$
				−2.76$\frac{|EN_{A}-EN_{B}{|}^{2}}{EN_{A}+EN_{B}}$ + 0.01$(EN_{A}+EN_{B}{)}^{4}$ −0.86
	3	0.753	0.709	2.33$|EN_{A}-EN_{B}|$ + 0.90$|EN_{A}-EN_{B}{|}^{2}$
				−0.17$(EN_{A}+EN_{B}{)}^{2}$$|EN_{A}-EN_{B}|$ + 0.005$(EN_{A}+EN_{B}{)}^{4}$
				−13.32${\left(\frac{|EN_{A}-EN_{B}|}{EN_{A}+EN_{B}}\right)}^{2}$ −0.85
